# Histological features of the gastric mucosa in children with primary bile reflux gastritis

**DOI:** 10.1186/1477-7819-10-27

**Published:** 2012-01-31

**Authors:** Yanyi Zhang, Xi Yang, Weizhong Gu, Xiaoli Shu, Ting Zhang, Mizu Jiang

**Affiliations:** 1 Department of Gastroenterology, Children's Hospital, Zhejiang University School of Medicine; Key Laboratory of Reproductive Genetics (Zhejiang University), Ministry of Education, Hangzhou 310003, P.R. China

**Keywords:** Bile reflux, Reflux gastritis, Bilirubin monitoring, Gastric mucosa, Children

## Abstract

**Background:**

Bile reflux is one of the primary factors involved in the pathogenesis of gastric mucosal lesions in patients with chronic gastritis; however, little is known about the exact histological features of bile reflux and its contributions to gastric mucosal lesions in this disease, especially in children with primary bile reflux gastritis (BRG). The aim of this study was to investigate the classic histological changes of the gastric mucosa in children with primary BRG.

**Methods:**

The Bilitec 2000 was used for 24 h monitoring of gastric bile in 59 children with upper gastrointestinal symptoms. The histological characteristics of the gastric mucosa were examined and scored.

**Results:**

Thirteen of the 59 patients had a helicobacter pylori infection and were excluded; therefore, 46 cases were included in this study. The positive rate of pathological duodenogastric reflux was significantly higher in patients with foveolar hyperplasia than those without foveolar hyperplasia; however, the rate was significantly lower in patients with vascular congestion than those without vascular congestion. The longest reflux time and the total percentage time of bile reflux were significantly lower in patients with vascular congestion than those without vascular congestion. A total of 9 types of histological changes were analyzed using a binary logistic regression. Foveolar hyperplasia and vascular congestion in the superficial layer became significant variables in the last step of the stepwise regression.

**Conclusions:**

Foveolar hyperplasia was associated with the severity of bile reflux, suggesting that it is a histological feature of primary BRG in children, while vascular congestion may be a protective factor.

## Background

In addition to gastric acid and Helicobacter pylori (HP), bile reflux is one of the primary factors involved in the pathophysiological processes leading to gastric mucosal lesions in patients with chronic gastritis; however, little is known about the exact histological features of bile reflux and its contributions to gastric mucosal lesions in this disease [1,2]. When duodenal contents reflux into the stomach for a short period during a physiological event, it causes few symptoms. However, duodenogastric reflux (DGR) becomes pathological when it is excessive or lasts for an extended period of time. Bile reflux gastritis (BRG) is due to an excessive reflux of bile, pancreatic and intestinal secretions into the stomach. The increased bile reflux may cause increased gastric mucosal injury. Primary BRG is defined as bile reflux-induced gastritis without a prior stomach surgery. Excessive DGR is very common in adults after gastric surgery, pyloroplasty, and cholecystectomy [3]. Dixon et al. [4] first reported the histological findings of bile reflux gastritis induced by DGR after a partial gastrectomy. They proposed a scoring system based on five characteristic histological features, including antral foveolar hyperplasia, vascular congestion, edema and smooth muscle fibers in the lamina propria and a paucity of inflammatory cells. The authors revealed significant associations between the histologic findings and hypochlorhydria and increased gastric bile acid concentration. However, a partial gastrectomy is not the only cause of BRG; similar changes have been reported in the intact stomach [5]. There are many reports of BRG in adults, but the histological features of gastric mucosa in children with primary BRG remain unclear [6,7]. The Bilitec 2000 method (Synectics Medical, Stockholm, Sweden), consisting of a fiber-optic probe connected to a portable bilimeter, is considered the most reliable of the currently available tools for detecting excessive bile reflux because it allows for 24 h continuous endoluminal monitoring of bilirubin exposure based on its spectrophotochemical properties [8,9]. In this study, ambulatory Bilitec 2000 with a fiber-optic spectrophotometer was used to monitor gastric bile reflux in 59 children with upper gastrointestinal symptoms, such as epigastric pain, nausea, vomiting, etc. The histological features of gastric mucosa were investigated to better understand the role of bile reflux in the pathophysiological processes of chronic gastritis in children.

## Methods

A total of 59 children (24 male and 35 female) with upper gastrointestinal symptoms were initially enrolled in this study from Oct. 2005 to Dec. 2008. Their median age was 10.6 years (range 3-17 years). Inclusion criteria in this study were as follows: (1) upper gastrointestinal symptoms, such as epigastric pain, nausea, vomiting, and belching, (2) no antibiotic or antacid use for 2 weeks before sampling, and (3) no corticosteroid or non-steroidal anti-inflammatory drugs (NSAIDs) use within 1 month of sampling. Exclusion criteria were as follows: history of gastric surgery or history of HP eradication, and psychomotor retardation. This study was approved by the ethics committee at Zhejiang University School of Medicine, China. Informed consent was obtained from patients or their relatives.

Gastroendoscopy (Olympus GIF-160, Japan) was performed in all patients, and 3 gastric antral biopsies were obtained to assess the changes in the histopathology. One was used to detect an HP infection by the rapid urease test (RUT), and the other two were immediately fixed in 10% formaldehyde and routinely processed. All patients underwent a 24 h ambulatory monitoring of the bilirubin absorbance in the gastric cavity using a Bilitec 2000 bile reflux monitor (Medtronic Instruments) two days after gastroendoscopy. Any drug that may inhibit gastric acid secretion, promote gastrointestinal motility, protect gastric mucosa or neutralize bile and antibiotics was stopped 2 weeks before monitoring. After calibrating the fiber-optic probe of the Bilitec 2000 in a dark, water-filled chamber, the probe was passed through the nares into the stomach and positioned 10-15 cm below the lower esophageal sphincter (LES) calculated by the following formula: the distance from nose to mid-point of the LES is (height of body × 0.252 + 5) cm. The catheter was then attached with adhesive tape to the patient's nose and cheek. The position of catheter was confirmed by radiography to ensure that transpyloric migration did not occur. Patients were allowed to perform their normal daily activities. Their parents were asked to record the time of food or fluid consumption and posture changes in a diary. During the recording time, only liquid meals, not interfering with Bilitec monitoring, were used. Patients were asked to avoid fruit juices, tea and coffee, whereas water intake was unrestricted.

The data from the Bilitec 2000 were downloaded and analyzed with standard, commercially available software (Esophogram, Gastrosoft Inc., Irving, Texas, USA). The bile reflux was confirmed if the value of bilirubin absorbance in the stomach was more than 0.14[10]. The parameters monitored included the number of bile reflux episodes, the number of bile reflux episodes that lasted for more than 5 min, the longest bile reflux time, and the total percentage time of bile reflux. Pathological bile reflux was diagnosed when the total percentage time of bile reflux more than 35.6% of the normal values from gastric bilirubin monitoring studies in healthy adults [11]. Based on the diagnostic criteria of bile reflux, the patients were divided into a bile reflux-positive group and a bile reflux-negative group.

All the serial sections (5 μm thick) prepared from formalin-fixed, paraffin-embedded biopsy samples underwent hematoxylin and eosin (HE) staining for conventional histological examination and modified Giemsa staining to detect for the presence of an HP infection. The presence of an HP infection was confirmed if RUT or Giemsa staining of the gastric antral mucosa were positive. Patients were excluded from this study if they had positive findings for an HP infection. The histological changes in gastric mucosa included the degree of inflammation, inflammatory activity, lymphatic follicle hyperplasia, antral atrophy, intestinal metaplasia, foveolar hyperplasia, interstitial edema, vascular congestion of superficial mucosa, and hyperplasia of smooth muscle fibers in the lamina propria. Each specimen without an HP infection was assessed for grading as normal, mild, moderate, or severe, based on Dixon's scoring system and was given a score of 0, 1, 2, and 3, respectively [4]. A senior pathologist performed the histological evaluation of gastric mucosa for all sections without the knowledge of other patient data.

### Statistical analysis

Results were recorded as the median (range) or number of cases. The parameters were compared between groups by the Wilcoxon rank-sum test using SPSS 16.0 statistical software. A chi-squared test was performed to analyze differences in the number of patients with pathological bile reflux and the different histological changes of the gastric mucosa. A binary logistic stepwise regression analysis was performed with pathological bile reflux as the independent variable (positive as 1 and negative as 0) and the degree of inflammation, inflammatory activity, lymphatic follicle hyperplasia, antral atrophy, intestinal metaplasia, foveolar hyperplasia, interstitial edema, vascular congestion of the superficial mucosa, and smooth muscle hyperplasia in the lamina propria as dependent variables. The results were considered statistically significant if the P value was less than 0.05.

## Results

### The degree of gastric bile reflux in different histological changes of the gastric antral mucosa

**Figure 1:**
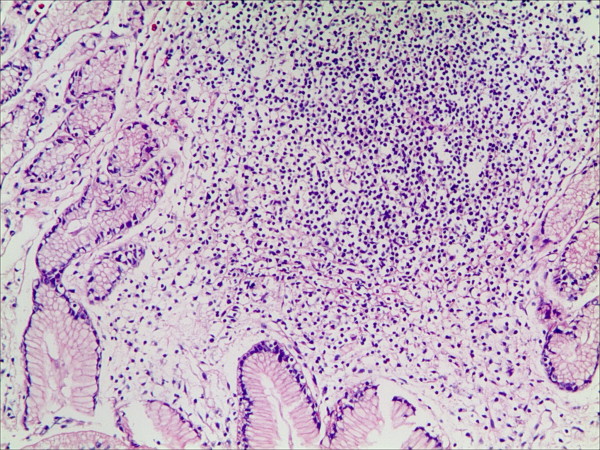
**Lymphatic follicle in the gastric mucosa in patients with bile reflux (HE staining, magnification 100×)**.

**Figure 2:**
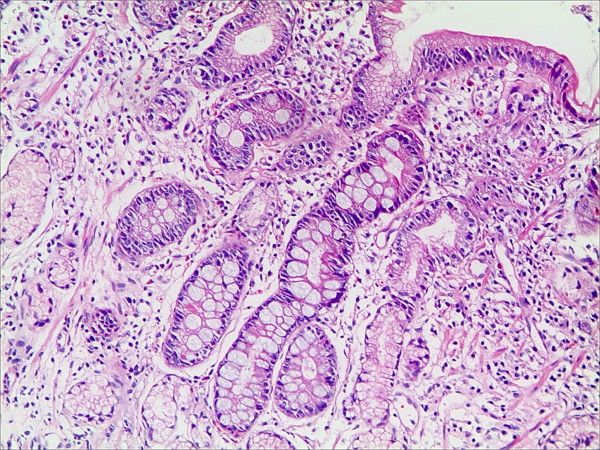
**Intestinal metaplasia in the gastric mucosa in patients with bile reflux (HE staining, magnification 100×)**.

**Figure 3:**
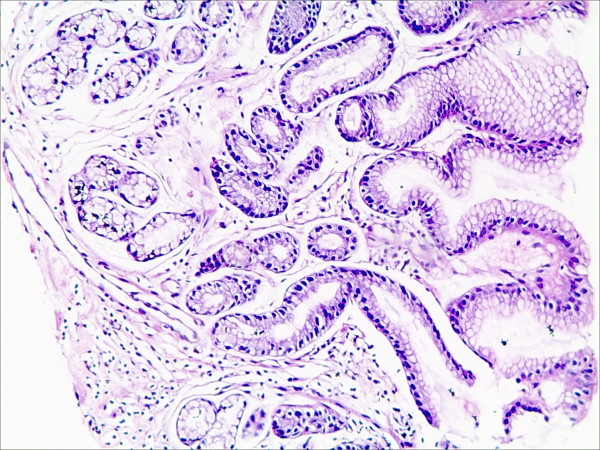
**Foveolar hyperplasia in the gastric mucosa in patients with bile reflux (HE staining, magnification 100×)**.

**Figure 4:**
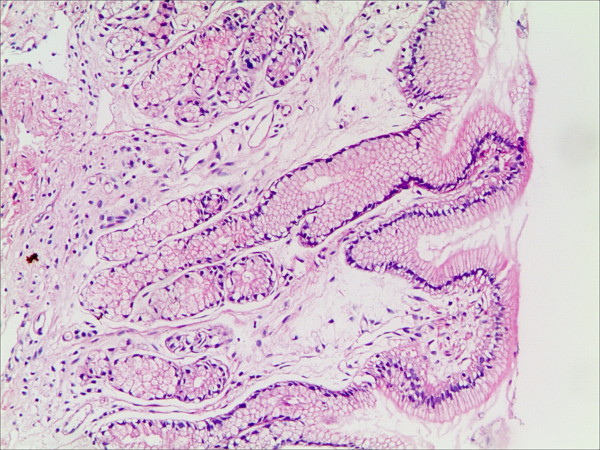
**Interstitial edema in the gastric mucosa in patients with bile reflux (HE staining, magnification 100×)**.

**Figure 5:**
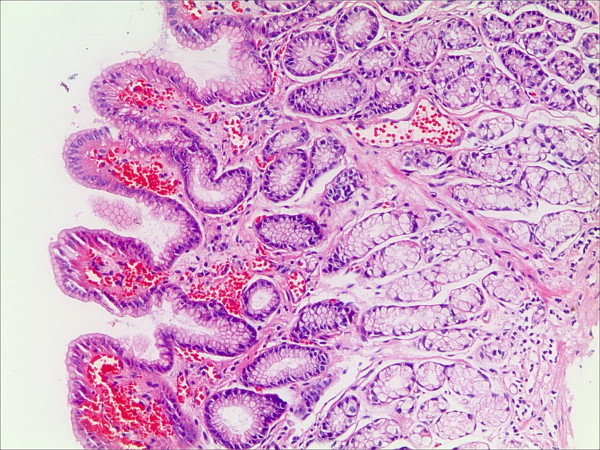
**Vascular congestion in the gastric mucosa in patients with bile reflux (HE staining, magnification 100×)**.

**Figure 6:**
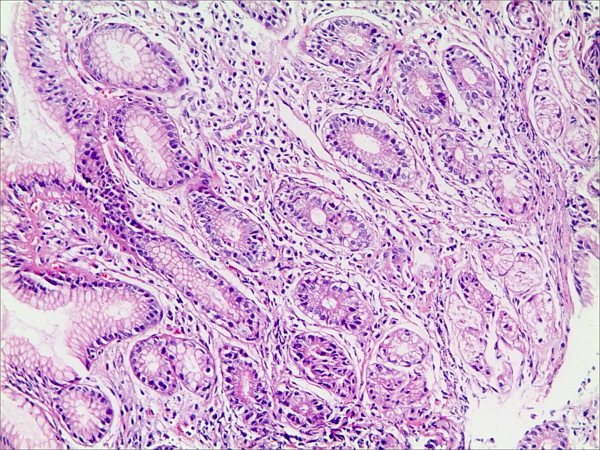
**Fibroproliferation in the gastric mucosa in patients with bile reflux (HE staining, magnification 100×)**.

Thirteen cases of 59 patients had HP infection; thus, according to the exclusion criteria, 46 patients were enrolled in this study. No gastric antral atrophy was found in these groups. The scores of different histological changes in the gastric antral mucosa and the parameters of bile reflux are shown in Table 1. Our results indicated that the longest reflux time and the total percentage time of bile reflux were significantly lower in cases with vascular congestion of the superficial mucosa than in those without vascular congestion [55(1-23) vs. 137(7-240), 22.8(0.9-55.1) vs. 35.2(3.5-82.8), respectively]. No significant differences in the bile reflux parameters were found for the other histological changes of the gastric mucosa. The gastric mucosal histological changes in patients with bile reflux are shown in Figure 1, 2, 3, 4, 5 and 6.

**Table 1:** Comparison of bile reflux parameters for the different histological types of the gastric antral mucosa [median (range)].

	Grade	n	number of bile reflux	number of bile reflux ≥ 5 min	longest time of reflux(min)	percentage of reflux time(%)
Degree of inflammation	0	5	82.0(17-150)	6.0(1-13)	117.0(14-141)	27.8(5.4-41.0)
	1	34	64.0(20-139)	9.0(0-23)	74.5(1-199)	25.4(0.9-82.8)
	2	7	66.0(38-264)	9.0(7-27)	63.0(28-240)	23.6(8.2-58.6)

Lymphatic follicle	0	40	66.5(17-264)	9.0(0-27)	74.5(1-199)	25.4(0.9-82.8)
	1	6	60.5(38-90)	7.5(3-11)	78.5(21-240)	30.4(7.5-58.6)

Intestinal metaplasia	0	43	61.0(17-150)	9.0(0-23)	69.0(1-240)	24.9(0.9-82.8)
	1	3	180(78-264)	25.0(7-27)	81.0(63-125)	39.3(20.7-55.1)

Foveolar hyperplasia	0	40	66.5(17-180)	8.5(0-27)	69.0(1-240)	24.5(0.9-82.8)
	1	6	64.0(23-264)	10.0(2-25)	116.0(34-223)	38.3(4.1-46.6)

Interstitial edema	0	23	70.0(17-150)	9.0(1-23)	82.0(5-240)	25.8(3.5-82.8)
	1	23	66.0(20-264)	8.0(0-27)	63.0(1-223)	24.9(0.9-55.1)

**Vascular congestion**	0	18	65.5(17-150)	9.0(1-23)	137(7-240)	35.2(3.5-82.8)
	1	28	66.5(20-264)	8.0(0-27)	55.0(1-223)**	22.8(0.9-55.1)*

Fibroproliferation	0	33	67.0(17-150)	8.0(0-20)	82.0(1-240)	24.8(0.9-82.8)
	1	13	66.0(27-264)	9.0(3-27)	63.0(28-220)	28.3(8.2-55.1)

* P < 0.05, ** P < 0.01, Wilcoxon rank-sum test comparing two or more groups

### DGR-positive ratio for different histological changes of the gastric antral mucosa

According to the diagnostic criteria of pathological DGR, 15 of the 46 cases were positive, and the other 31 cases were negative. As shown in Table 2, the positive rate of pathological DGR was significantly higher in cases with foveolar hyperplasia (5/6) than in those without foveolar hyperplasia (10/40); however, the rate was significantly lower in cases with vascular congestion of superficial mucosa (5/28) than in those without vascular congestion (10/18). No significant differences in the pathological DGR positive rate were found in patients with other histological changes of the gastric mucosa.

**Table 2:** Number of DGR-positive cases for the different histological changes of the gastric antral mucosa

	grades	Pathological duodenogastric reflux		total	**χ**^**2**^
				
		negative (n = 31)	positive(n = 15)		
Degree of inflammation	0	3	2	5	
	1	24	10	34	
	2	4	3	7	0.617

Lymphatic follicle	0	28	12	40	
	1	3	3	6	0.950

Intestinal metaplasia	0	30	13	43	
	1	1	2	3	1.694

Foveolar hyperplasia	0	30	10	40	
	1	1	5	6	8.079*

Interstitial edema	0	14	9	23	
	1	17	6	23	0.890

Vascular congestion	0	8	10	18	
	1	23	5	28	7.086*

Fibroproliferation	0	23	10	33	
	1	8	5	13	0.282

* P < 0.01, chi-squared test comparing two or more groups

### Results of binary logistic regression analysis

Nine types of histological changes in the gastric mucosa were analyzed using a stepwise regression, as shown in Table 3; however, no cases had inflammation activity or antral atrophy. Foveolar hyperplasia and vascular congestion of the superficial mucosa were the significant variables in the last step of the regression. These 2 variables were significantly correlated with bile reflux: foveolar hyperplasia was a risk factor for reflux, and vascular congestion was as a protective factor.

**Table 3:** Results of the binary logistic stepwise regression analysis.

	B	SE	wald	P	Exp(B)
Degree of inflammation	0.481	0.952	0.255	0.613	1.618
Lymphatic follicle	1.639	1.608	1.039	0.308	5.150
Interstitial metaplasia	3.274	2.009	2.657	0.103	26.417
**Foveolar hyperplasia**	3.593	1.636	4.825	0.028	36.343
Interstitial edema	0.145	1.117	0.017	0.897	1.156
**Vascular congestion**	-3.915	1.449	7.302	0.007	0.020
Fibroproliferation	0.322	1.145	0.079	0.779	1.380
Constant	-0.506	0.834	0.369	0.544	0.603

a. Variable(s) entered on step 1: inflammation, lymphatic, metaplasia, hyperplasia, edema, congestion, fribroproliferation.

## Discussion

The gastric mucosa lesions caused by bile reflux are common in the postoperative stomach. Recently, histopathological changes in the gastric mucosa in patients with primary BRG have received more attention [6,7]. Bile reflux is thought to be associated with chronic inflammation of the gastric mucosa, lamina propria edema, foveolar hyperplasia, antral atrophy, and intestinal metaplasia [1]. In 1986, Dixon et al. [4] established a scoring system for the pathomorphisms of the postoperative stomach. Postoperative BRG could be accurately diagnosed according to its characteristic histopathological changes, such as foveolar hyperplasia, edema and congestion of the lamina propria, and a smaller degree of inflammatory cellular infiltration [1,12]. However, the histological features of primary BRG remain controversial. Wang et al. [13] reported that foveolar hyperplasia existed in both the gastric corpora and gastric antrum with mild chronic inflammation in a rat model of long-term bile reflux, which is consistent with the pathological features of reflux gastritis. Bile reflux is known to be associated with the development of intestinal metaplasia in adult [5,12], but the relationship between bile reflux and intestinal metaplasia in children remains unclear.

Bile reflux has been suggested as a cause of gastropathy in pediatric gastroenterology textbooks and in comprehensive reviews of gastritis in children [14,15]. It is also unclear whether primary BRG in children has characteristic histological changes in gastric mucosa. Pashankar et al. [7] identified all of the classic histopathologic features of chemical gastropathy in 21 children: 19 patients had foveolar hyperplasia, 20 patients had vascular congestion, 16 patients had lamina propria edema, and 16 patients had smooth muscle hyperplasia. They concluded that bile reflux, gastroesophageal reflux disease, and the use of medications, such as NSAIDs, may affect the pathogenesis of chemical gastropathy in children. Based on Dixon's scoring system, no scores of 2 or 3 grade of gastric mucosa were observed in the different histological types, except for the degree of inflammation. It is speculated that the severity of bile reflux was lower than in the cases reported by Dixon et al., which involved secondary BRG. Nevertheless, the relationship between the degree of bile reflux and the histological changes in the gastric mucosa remains unknown. Our results indicated that the longest reflux time and the total percentage time of bile reflux were significantly lower in cases with vascular congestion of the gastric mucosa than in those without vascular congestion.

Pathological DGR was confirmed using ambulatory intragastric bile monitoring by the Bilitec 2000 optoelectronic device. Although absorbance values recorded in our patients could not be compared to age-matched controls for ethical reasons, one study had reported the 95th percentile values of bile exposure at absorbance to be 0.14 in healthy Chinese adults [11]. According to this study, patients had pathological DGR because they had values that exceeded the 95th percentile for the total percentage time of bile exposure at 0.14 to 35.6% the control threshold. The positive rate of pathological DGR was significantly higher in cases with foveolar hyperplasia than in those without foveolar hyperplasia; however, it was significantly lower in cases with vascular congestion of the superficial mucosa than in those without vascular congestion. This study revealed that the degree of bile reflux was severe in patients with foveolar hyperplasia and mild in patients with vascular congestion. Interestingly, only 3 of the 46 patients had intestinal metaplasia, which was not similar to other studies in adults [1]. In fact, intestinal metaplasia is a type of adaptive change of the gastric mucosa that occurs when the local microenvironment of gastric mucosa changes to the microenvironment of the intestinal tract [14,16]. These results are expected to be found more frequently in future studies with larger sample sizes, thus providing a more comprehensive understanding of the characteristics of these lesions.

To investigate the relationship between pathological DGR and histological changes, a binary logistic regression analysis was performed for the 9 types of histological characteristics in the gastric antral mucosa as mentioned in the methods. Foveolar hyperplasia and vascular congestion in the superficial layer was the significant variables in the last step of the stepwise regression. Foveolar hyperplasia was a risk factor for patients with bile reflux, and vascular congestion was a protective factor. Gastric mucosal damage caused by bile reflux induces mast cell degranulation and a release of vasoactive mediators, such as histamine, leading to vascular congestion and lamina propria edema [17]. The vascular congestion of the superficial mucosa was thought to be an adaptive change in the gastric mucosa with protective effects. The blood supply is important in maintaining the normal function of the cells, including cell repair and replacement. Expansion of the superficial vessels increases mucosal blood flow, which may quickly remove harmful substances and is beneficial for mucosal regeneration after an injury. This cell-protective effect may be related to the secretion of prostaglandins.

## Conclusions

In conclusion, foveolar hyperplasia is associated with the degree of bile reflux and may serve as the characteristic histological change of primary BRG in children. Our study confirmed the negative correlation between vascular congestion of the superficial mucosa and bile reflux gastritis.

## Abbreviations list

BRG: bile reflux gastritis; DGR: duodenogastric reflux; HE: hematoxylin and eosin; HP: *Helicobacter pylori; *RUT: rapid urease test; LES: lower esophageal sphincter; NSAIDs: non-steroidal anti-inflammatory drugs.

## Competing interests

The authors declare that they have no competing interests.

## Authors' contributions

MJ carried out design of the study. YZ and MJ conducted the experiments, analyzed data, and drafted the manuscript. WG and XS performed the pathological diagnosis. YZ, XY, TZ, and XS were involved in the collection of gastric mucosa, data analysis, and reviewed the paper. All authors read and approved the final manuscript.
